# Insights into the molecular evolution of oxytocin receptor ligand binding

**DOI:** 10.1042/BST20120256

**Published:** 2013-01-29

**Authors:** Johannes Koehbach, Thomas Stockner, Christian Bergmayr, Markus Muttenthaler, Christian W. Gruber

**Affiliations:** *Medical University of Vienna, Center for Physiology and Pharmacology, Schwarzspanierstr. 17, A-1090 Vienna, Austria; †Departments of Chemistry and Cell Biology, The Scripps Research Institute, 10550 North Torrey Pines Road, La Jolla, CA 92037, U.S.A.

**Keywords:** arginine vasopressin, binding, GPCR (G-protein-coupled receptor), homology model, OT (oxytocin), vasotocin, AVP, arginine vasopressin, CTR, cephalotocin receptor, GPCR, G-protein-coupled receptor, ICL, intracellular loop, OT, oxytocin, OTR, oxytocin receptor, TM, transmembrane, VT, vasotocin

## Abstract

The design and development of selective ligands for the human OT (oxytocin) and AVP (arginine vasopressin) receptors is a big challenge since the different receptor subtypes and their native peptide ligands display great similarity. Detailed understanding of the mechanism of OT's interaction with its receptor is important and may assist in the ligand- or structure-based design of selective and potent ligands. In the present article, we compared 69 OT- and OT-like receptor sequences with regards to their molecular evolution and diversity, utilized an *in silico* approach to map the common ligand interaction sites of recently published G-protein-coupled receptor structures to a model of the human OTR (OT receptor) and compared these interacting residues within a selection of different OTR sequences. Our analysis suggests the existence of a binding site for OT peptides within the common transmembrane core region of the receptor, but it appears extremely difficult to identify receptor or ligand residues that could explain the selectivity of OT to its receptors. We remain confident that the presented evolutionary overview and modelling approach will aid interpretation of forthcoming OTR crystal structures.

## Introduction

The neurohypophyseal nonapeptides OT (oxytocin) and AVP (arginine vasopressin) play an important role in many physiological functions through GPCR (G-protein-coupled receptor) signal transduction. In humans and other mammalian species, OT and AVP target the OTR (oxytocin receptor) and the three vasopressin receptors V1aR, V1bR and V2R [1]. Peripherally, OT acts as a hormone that triggers uterine smooth muscle contraction during childbirth, facilitates milk ejection for breastfeeding and is involved in male ejaculation. Centrally, it functions as a neurotransmitter where it is involved in complex social behaviour, maternal care, stress and anxiety [2]. AVP regulates fluid balance and blood pressure and is mainly involved in memory, learning, stress regulation and aggressive behaviour (summarized in [1]). Although OT and AVP elicit distinct physiological functions, they display a certain degree of cross-reactivity, which is caused by the high ligand similarity (OT and AVP only differ by two residues) and the high extracellular homology (~80%) between the OT and AVP receptors. Contrary to many other signalling systems, selectivity is not achieved via the ligands, but via interplay of factors including receptor up- or down-regulation, release of specific ligand-degrading enzymes, local ligand production and receptor clustering. In terms of drug development, the extracellular receptor homology represents a major hurdle since a lack of selectivity can cause undesired side effects [3,4]. To address the need for receptor-selective ligands, we decided to review the molecular evolution of the OT/AVP ligand–receptor system to gain insight into the mechanism of receptor–ligand recognition and binding.

## Evolutionary aspects of the OT–vasopressin signalling system

OT/AVP-related peptides have been found in a wide range of species and are thought to have evolved via gene duplication from the ancestral VT (vasotocin) peptide some 600 million years ago [5]. VT-like peptide hormones have been found in non-mammalian vertebrates, fish, mammals, molluscs, annelids, arthropods and several insects, including social ants [6]. All members of the OT, AVP and VT peptide family share high sequence similarity, namely an N-terminal six-residue ring, formed by a disulfide bond between the two cysteine residues at positions 1 and 6, and a flexible C-terminal three-residue tail with a highly conserved Pro^7^ and a glycine amide at position 9 (CXXXXCPXG). Residue variability is observed at positions 2–5 and at position 8 [3]. These amino acid variations are presumably responsible for species-selective recognition, binding and activation of the different receptors. Although the four human receptors display high extracellular sequence similarity, there exists low inter-species receptor correlation reflected by the fact that many ligands selective in rat or mice are not selective in humans [7]. Hence it was of interest to us whether inter-species differences of the individual native ligands could be correlated to receptor sequence variations and vice versa. We hypothesized that a detailed molecular understanding of recognition, binding and activation of OT and AVP receptors by their native ligands could assist the design and development of novel selective ligands.

## OTR sequence comparison of residues Arg^34^, Phe^103^, Tyr^209^ and Phe^284^

**Figure 1:**
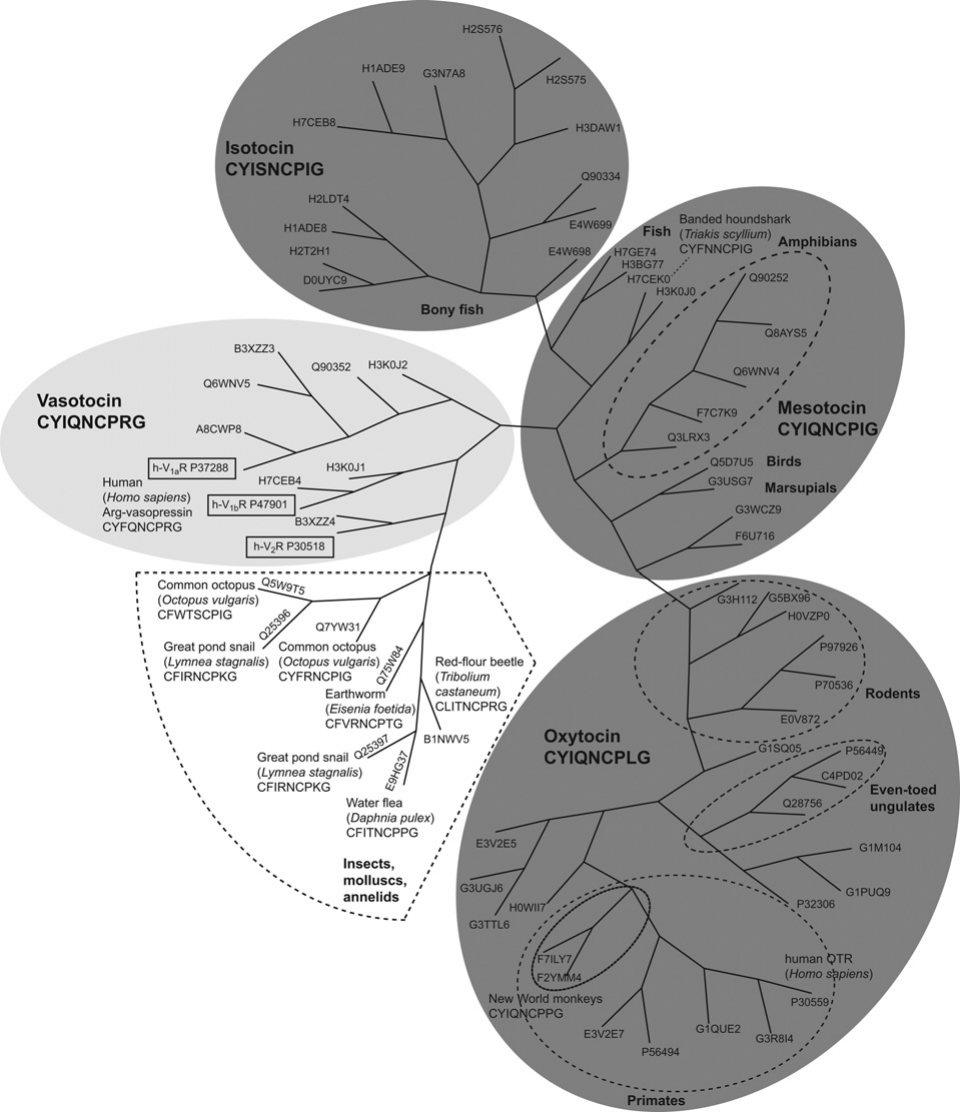
Evolution of the OTR and its endogenous ligands A phylogenetic tree consisting of 69 OT, OT-like, VT and the human vasopressin receptors and their respective ligands is shown and has been prepared by sequence alignment using ClustalW. Receptor clusters for OT, isotocin and mesotocin are indicated in dark grey and VT/vasopressin receptors (presumably the ancestral group) are highlighted in light grey. Receptors of species within the same class or order are highlighted by broken lines. The UniProtKB entry numbers of the receptors are shown at the end of each branch. During production of the present review, two research groups independently reported the discovery and functional characterization of an OT/VT-like signalling system in *Caenorhabditis elegans* [36,37]. The two nematocin receptors and their peptide ligands are involved in the nematode's gustatory associative learning and reproductive behaviour. In contrast with humans and other animals, these nematodes produce an undecapeptide that is only functional at full length. It contains the typical six-residue N-terminal ring, but has an additional two residues at its C-terminus. The evolutionary perspective of this modification, its influence on ligand binding and the phylogenetic relationship of the receptors have yet to be established.

A total of 69 known receptor sequences of OT, OT-like, VT and human AVP GPCRs with their native ligands were selected from a wide range of species for sequence comparison. The phylogenetic tree analysis based on these sequences was performed with ClustalW and Figure 1 illustrates the comparison of the ligand sequence variation with the receptor sequence evolution. Consistent with the high receptor sequence similarity across closely related species (e.g. bony fish, amphibians or primates), it was found that the respective ligands within these clusters were also highly conserved. This is in agreement with the evolution of OT/AVP and related nonapeptides [9,10]. Residues believed to be responsible for OT–AVP ligand–receptor binding were analysed and their degree of conservation was compared.

**Figure 2:**
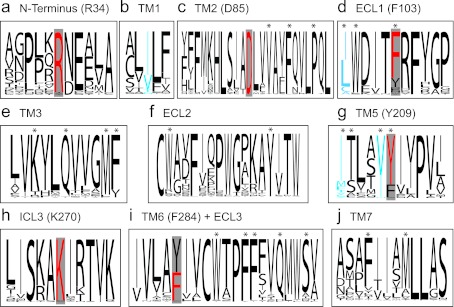
Molecular comparison of OTR residues involved in ligand binding Sequence logos of short sections of aligned receptors in key positions that are involved in ligand recognition, binding and receptor functionality for the human OTR as well as residues found in contact with the ligand, obtained from the GPCR ligand co-crystal after structural super-positioning, are shown (Supplementary Table S1 at http://www.biochemsoctrans.org/bst/041/bst0410197add.htm). The sections (solid boxes) include the N-terminal residue Arg^34^ (**a**), Asp^85^ in TM1 (**b**) in TM2 (**c**), Phe^103^ in the ECL (extracellular loop) 1 (**d**), TM3 (**e**), ECL2 (**f**), Tyr^209^ in TM5 (**g**), Lys^270^ in ICL3 (**h**), Phe^284^ in TM6 (**i**) and TM7 (**j**). Numbering of residues is based on the human OTR sequence. Residue positions of importance, on the basis of previous biochemical studies, are highlighted by grey boxes and OT receptor residues are highlighted in red. Common GPCR–ligand interacting residues (highest frequency show residues Gln^119^, Met^123^, Ile^204^, Val^208^, Trp^288^, Phe^291^, Phe^292^, Gln^295^ and Met^315^; numbering according to human OTR; Figure 3 and Supplementary Table S1) are labelled with an asterisk and residues that correlate with the respective ligand sequence (i.e. Ile^49^, Leu^98^, Ile^204^ and Val^208^; numbering according to human OTR) are coloured in blue.

Previous studies determined that the residues Arg^34^, Phe^103^, Tyr^209^ and Phe^284^ were important for ligand binding and selectivity at the human OTR [11–15]. Additionally, Asp^85^ and Lys^270^ are involved in receptor signalling [16]. Interestingly, all OT and OT-related receptors share the Asp^85^ residue in the TM (transmembrane) domain 2 and the Lys^270^ residue in ICL (intracellular loop) 3 (Figures 2c and 2h), and these residues were reported to be important for receptor activation [17,18]. Particularly, the Asp^85^ is also conserved in other class A GPCRs and is thought to play a more general role in receptor–G-protein signalling [18].

The N-terminal Arg^34^ is highly conserved (only three receptors have a different residue in that position: Asp^34^ in chicken OTR, Tyr^34^ in cephalotocin receptor CTR2 and Val^34^ in conopressin receptor LSCPR1; see Figure 2a), indicating its importance for ligand binding [11]. Phe^103^ was demonstrated to be important for ligand selectivity in the OTR and is thought to interact with the residue at position 8 of the peptide ligand [16]. OT receptors with a phenylalanine in that position generally bind native ligands that have either a leucine or an isoleucine residue at position 8 (in contrast to Arg^8^ of AVP, with human V1aR containing a tyrosine at corresponding position 115). Exceptions are the OT-like peptide from the New World monkeys *Saimiri sciureus* and *Callithrix jacchus* as well as inotocin from the water flea *Daphnia pulex*, which have a proline at position 8. Interestingly, the receptors of *C. jacchus* and *D. pulex* have a hydrophobic phenylalanine at corresponding position 103 whereas the receptor of *S. sciureus* has a hydrophilic tyrosine in that position, indicating that a direct interaction of this residue in the receptor and the residue at position 8 of the ligand may not be present in all OT receptor–ligand pairs, since the interaction of the polar tyrosine and the hydrophobic proline residues is much weaker compared with the hydrophobic phenylalanine–proline interaction. Further examples of ligand–receptor variation in position 103 of the receptor and position 8 of the ligand include the red flour beetle *Tribolium castaneum* (tyrosine in receptor, arginine in ligand), the earthworm *Eisenia foetida* (histidine in receptor, threonine in ligand), the great pond snail *Lymnea stagnalis* (isoleucine in receptor and ligand) and the common octopus *Octopus vulgaris* (valine in receptor, isoleucine in ligand). Tyr^209^ and Phe^284^, located in the TM region are two other important residues for ligand–receptor binding [16]. In the human receptor, Tyr^209^ and Phe^284^ interact with residues at positions 2 and 3 of OT [15,16]. Tyr^209^ is highly conserved among all OT-like receptors, being a tyrosine in most sequences or another aromatic residue (Figure 2g). All native peptide ligands contain an aromatic residue (tyrosine or phenylalanine) in position 2 and a hydrophobic or aromatic residue in position 3 (isoleucine, phenylalanine, valine or tryptophan) [3] indicating that this ligand–receptor interaction may indeed be conserved throughout evolution. This is also true for VT and human AVP receptors that we analysed; the receptors contain a tyrosine in position 209 and their respective ligands contain a tyrosine in position 2 and a phenylalanine or isoleucine in position 3 (Figure 1). Accordingly, the residue at position 284 of the receptor is, in most instances, aromatic (phenylalanine or tyrosine) and the only exception is the CTR1 from *O. vulgaris* that contains a cysteine in that position (Figure 2i). Again, these residues are possibly involved in hydrophobic receptor–ligand interactions. The importance of N-terminal hydrophobicity of the ligands is further supported by the identification of the superagonist desamino-OT, where the deletion of the N-terminal amine led to a more hydrophobic and potent OT analogue [19]. The analysis of the ligand–receptor evolution with respect to the residues at positions 34, 103, 209 and 284 of OT-like receptors is in agreement with previous studies (summarized in [16]), but it is likely that these molecular contacts may not be present in all native ligand–receptor pairs.

To gain further insight into the molecular interaction of OT and its receptors, we created a homology model of the human OTR based on recently published GPCR structures. Based on the information from various ligand co-crystal structures of human or mammalian GPCRs [20–30], one may anticipate that not only certain residues, but also the overall three-dimensional receptor architecture of the human OTR plays a significant role in ligand–receptor interactions. Residues that interact with GPCR ligands were extracted from crystal structures and were mapped on to the homology model of the OTR as discussed in the next paragraph.

## Ligand–GPCR interaction: lessons learned from recent crystal structures

**Figure 3:**
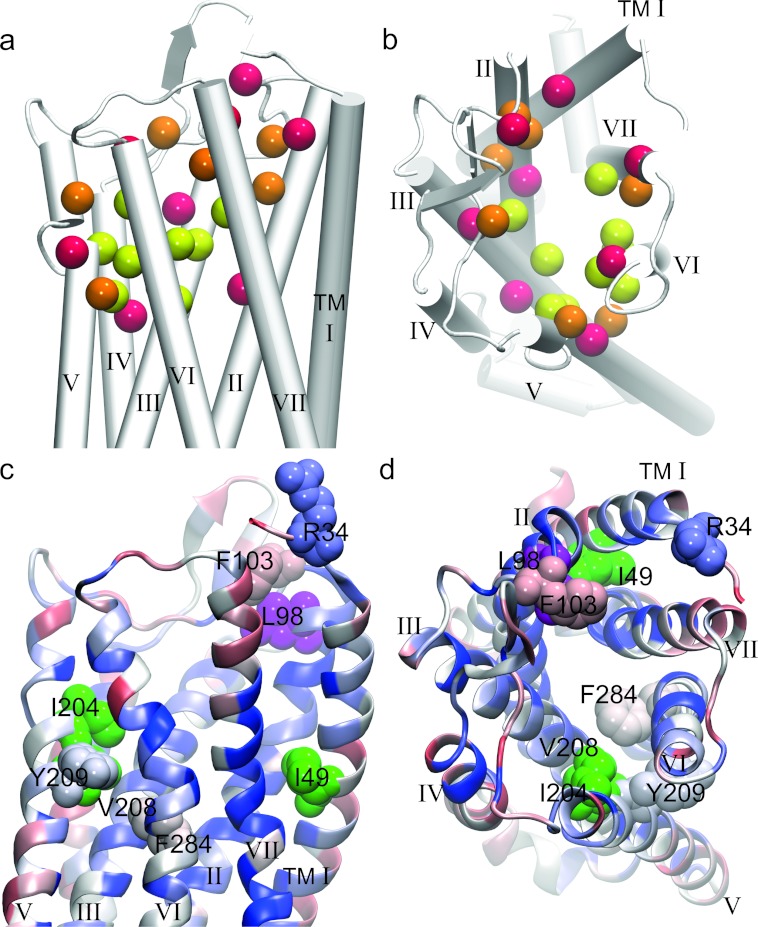
Mapping of GPCR ligand-binding sites on to the structural model of the human OTR Sequences of the OTR and the opioid receptor were aligned with muscle [34] and a model of the human OTR was built using MODELLER v9.8 [35] applying the automodel procedure, of the mouse μ-opioid receptor crystal structure as a template (PDB ID: 4DKL) [21]. (**a** and **b**) Residues that interact with GPCR ligands have been extracted from crystal structures and are listed in Supplementary Table 1 at http://www.biochemsoctrans.org/bst/041/bst0410197add.htm. The residues that interact with a GPCR ligand have been mapped on to the homology model of the human OTR. The β-carbon atoms of these residues are shown as van der Waals spheres. Colour coding highlights interaction frequency: yellow, >75%, orange, 25–75% and red, <25%. Residues displaying high interaction frequency line the surface of the binding pocket. (**c** and **d**) Residue conservation of OTRs: per residue sequence conservation of OT sequences, analysed by sequence alignment, are visualized in the framework of the three-dimensional structure of the OTR model. The yellow spheres show the β-carbon atoms of the residues interacting with ligand. The colour-coding of the OTR model highlights the per residue conservation ranging from dark blue (identical) to red (opposite type of amino acid). Conservation analysis was carried out using ClustalW. The four residues (Arg^34^, Phe^103^, Tyr^209^ and Phe^284^) of the OTR known to affect binding of the native ligands are shown in space filling. Sequences excluded from the similarity analysis owing to incompletion are: G3US67, G5BX96, G3H112 and B5UA19. The following structures were used for the interaction analysis: 2RH1, 2Y00, 2Y04, 3D4S, 3EML, 3OAX, 3OE6, 3PBL, 3SN6, 3UON, 3V2Y, 4DAJ, 4DJH, 4DKL, 4EA3, 4EJ4, 2VT4, 3P0G, 3PDS, 3PWH, 3RZ4 and 3V2W. TM α-helices are shown as grey cylinders or helix cartoons and are labelled from TMI–VII. Residues that correlate with the respective ligand sequence (i.e. Ile^49^, Leu^98^, Ile^204^ and Val^208^; numbering according to human OTR) are shown in magenta and green respectively.

Ligands are recognized by the extracellular region of the receptor and interact with residues in the three-dimensional environment of the receptor TM domain. This has been observed for orthosteric ligands that interact with class A GPCRs in recent crystal structures [30]. With the aim of understanding the binding mode of OT to its receptor in the context of its three-dimensional architecture, we developed a homology model of the human OTR on the template of the mouse μ-opioid receptor crystal structure with the intention of identifying whether and which residues are oriented towards the binding pocket and interact with the ligand. The mouse μ-opioid receptor was selected owing to its high sequence similarity, high resolution and canonical receptor conformation. We analysed the publicly available crystal structures (for details see caption of Figure 3) and determined the contacts of the OTR side chains with the respective ligand (Supplementary Table S1 at http://www.biochemsoctrans.org/bst/041/bst0410197add.htm) by overlaying the GPCR crystal structures with the OTR and then transferring each ligand from the crystal structures into the OTR model. The observed frequency of residue–ligand contacts (i.e. the number of structures in which a direct interaction of the receptor residue with the ligand was observed) was mapped on to the OTR model. With this approach we were able to define a common binding site, deep within the vestibule of the receptor structure that is shared by all GPCR ligands (agonists and antagonists) that were used for this analysis. The positions with the highest frequency of direct contact with ligands include Gln^119^ and Met^123^ in helix 3, Ile^204^ and Val^208^ in helix 5, Trp^288^, Phe^291^, Phe^292^ and Gln^295^ in helix 6 and residue Met^315^ in helix 7 (numbering according to human OTR). The α-carbon atoms of all these residues are within 16 Å (1 Å=0.1 nm) from each other. The side chains are all orientated towards the ligand-binding vestibule and many of them are in direct contact with each other. Residues at the rim of the vestibule displayed lower interaction frequency and it appears that this strongly depends on the size and orientation of the ligand (Figures 3a and 3b). Of note, we did not distinguish between different types of ligand such as agonist or antagonist.

Contrary to what we expected, the four residues described in the literature to be important for human OT ligand–receptor interaction (Arg^34^, Phe^103^, Tyr^209^ and Phe^284^) are not within the group of frequently occurring interacting residues. Arg^34^ and Phe^103^ are located at the rim of the vestibule, above the common interacting residues, on the extracellular side, whereas Tyr^209^ and Phe^284^ (TM5 and TM6 respectively) are located below the common binding site. Phe^284^ is located below Trp^288^ in the centre of the TM core region. Phe^284^ can form π interactions with Trp^288^ and is believed to contribute to orienting the side chain of Trp^288^, which faces towards the proposed ligand-binding pocket and was found to be in contact with ligands in high frequency in GPCR structures. The four residues (Arg^34^, Phe^103^, Tyr^209^ and Phe^284^) that are important for OT binding are located below and above the site in which the model structure provided information on the interaction between ligands and GPCRs with high frequency. However, the ligands in the analysed crystal structures were all small organic molecules, significantly smaller than the nonapeptides in this study. The size of the nonapeptides would allow them to interact simultaneously with Tyr^209^ and Phe^284^ at the bottom of the ligand binding pocked and with Arg^34^ and Phe^103^ on the extracellular side. It is therefore very likely that OT binds to a common binding site that is located in between those four residues (Arg^34^, Phe^103^, Tyr^209^ and Phe^284^) as proposed in the OTR model. Recently, the first crystal structure of a peptide-agonist-bound GPCR, namely the neurotensin receptor 1 has been reported [30a]. In agreement with our finding that the binding pocket involves residues in the common binding motif as well as on the extracellular side, White et al. [30a] found that neurotensin (residues 8–13) binds closer to the surface of its receptor compared with other small-molecule agonists.

The research groups of Slusarz et al. [31] and Fanelli et al. [18] have previously studied the interaction of human OT with its receptor by molecular dynamics simulation and identified a number of residues, mainly within the TM region of the receptor, considered important for interaction with the ligand. A comparison of these studies with our approach to map GPCR ligand-binding residues on to the homology model of the human OTR identified the common residues Lys^116^, Gln^119^, Met^123^ and Gln^295^ that display a high frequency contact in our model (Figure 3 and Supplementary Table S1). Following the analysis of the human OTR model and the common GPCR ligand-binding sites, we return now to the receptor–ligand sequence comparison in order to discuss the significance of the residues that were identified in our modelling approach.

## Comparison of the structural model and the OTR–ligand sequence pairs

On the basis of the three largest clusters of receptor–ligand evolution (Figure 1) and with a focus on the residues forming the common binding site in GPCR–ligand structures (Figure 3 and Supplementary Table 1), we compared the sequences of OT, isotocin and mesotocin receptors and their respective ligands (Figure 2). Residues of the seven TM helices that were oriented towards the ligand-binding site were found to be more conserved compared with the membrane exposed residues. Interestingly, the degree of conservation was unevenly distributed: helices 2, 3 and 6 were the most conserved, whereas residues in helix 7 displayed the largest variability. This was also observed in the comparison of the human OTR with the human V1aR and in the analysis of the binding pockets of the opioid receptors (T. Stockner, unpublished work), whereby interacting residues of TM6 are highly conserved and seem to be important for efficacy, whereas ligand-interacting residues of TM7 are highly variable and determine the selectivity of the receptor for its ligand [20,25]. Residues in TM7 show particular variation in the mesotocin receptor sequences, but we were not able to correlate these changes with variations in the ligand sequence. Four residues 49, 98, 204 and 208 in the receptor displayed some degree of correlation with the respective ligand and these residues are highlighted in Figures 2(c), 2(d), 2(g), 3(c) and 3(d).

The sequence of residue 98 (shown in magenta in Figures 3c and 3d) correlates with changes in the ligand: all analysed receptors have a non-polar aliphatic amino acid at this position (isoleucine or leucine) and the same amino acid in position 8 of the ligand. Residues in positions 49, 204 and 208, i.e. Ile^49^, Ile^204^ and Val^208^ in OT and mesotocin receptors (shown in green in Figures 3c and 3d) and Val^49^, Ile^204^ or Met^204^ and Ile^208^ in isotocin receptors show correlation with residue 4 in the nonapeptide sequence, which is either a glutamine in OT and mesotocin or a serine in isotocin. Since the amino acid changes in the receptor are conservative, i.e. amino acid type and hydrophobicity remain almost unchanged, we believe that these differences mainly affect residue size and could potentially result in a gentle variation in the size of the binding pocket.

## Summary and conclusion: only a ligand-bound structure can tell…

Biochemical studies with the human OT receptor and its native ligand have identified four residues that are important for ligand binding and recognition, namely Arg^34^, Phe^103^, Tyr^209^ and Phe^284^ (summarized in [16]). In the present review, we compared 69 OT, OT-like and VT receptor sequences to gain further insight into OT ligand binding. We utilized an *in silico* approach to map the common ligand interaction sites of recently published GPCR structures to a model of the human OT receptor and compared the interacting residues within different receptor sequences. Our analysis suggests the existence of a binding site for OT peptides within the TM core region. Previous evolutionary studies of OTRs by van Kesteren and Geraerts [32] and Cho et al. [33] pointed out a number of conserved residues in TM2, TM3, TM4, TM6 and TM7 that may be important for ligand binding, but their analysis included only a few different receptor sequences. It is evident from our comparison that TM7 displays the greatest sequence variability, which might be the source for interspecies selectivity. This helix also experiences the biggest structural movements when the receptor is activated and is certainly important for G-protein signalling [30]. Although this review brings together empirical data, molecular sequence comparison and homology modelling, there is still a lack of structure–activity and mutagenesis studies to propose a working OTR model that could explain selectivity differences observed in binding studies. We conclude that only a ligand-bound crystal structure of OT and its receptor will be able to shed light on to the mechanism of interaction and provide the means for future design of novel and selective ligands.
